# Neuroimaging of autobiographical memory in dementia with Lewy bodies: a story of insula

**DOI:** 10.1093/braincomms/fcae272

**Published:** 2024-08-20

**Authors:** Alice Tisserand, Frédéric Blanc, Candice Muller, Hélène Durand, Catherine Demuynck, Alix Ravier, Léa Sanna, Paulo Loureiro de Sousa, Anne Botzung, Mary Mondino, Nathalie Philippi

**Affiliations:** ICube Laboratory UMR 7357 and FMTS (Fédération de Médecine Translationnelle de Strasbourg), IMIS Team and IRIS Platform, University of Strasbourg and CNRS, 67000 Strasbourg, France; CM2R (Research and Resources Memory Centre), Geriatric Day Hospital and Neuropsychology Unit, Geriatrics Department and Neurology Service, University Hospitals of Strasbourg, 67000 Strasbourg, France; ICube Laboratory UMR 7357 and FMTS (Fédération de Médecine Translationnelle de Strasbourg), IMIS Team and IRIS Platform, University of Strasbourg and CNRS, 67000 Strasbourg, France; CM2R (Research and Resources Memory Centre), Geriatric Day Hospital and Neuropsychology Unit, Geriatrics Department and Neurology Service, University Hospitals of Strasbourg, 67000 Strasbourg, France; CM2R (Research and Resources Memory Centre), Geriatric Day Hospital and Neuropsychology Unit, Geriatrics Department and Neurology Service, University Hospitals of Strasbourg, 67000 Strasbourg, France; CM2R (Research and Resources Memory Centre), Geriatric Day Hospital and Neuropsychology Unit, Geriatrics Department and Neurology Service, University Hospitals of Strasbourg, 67000 Strasbourg, France; CM2R (Research and Resources Memory Centre), Geriatric Day Hospital and Neuropsychology Unit, Geriatrics Department and Neurology Service, University Hospitals of Strasbourg, 67000 Strasbourg, France; CM2R (Research and Resources Memory Centre), Geriatric Day Hospital and Neuropsychology Unit, Geriatrics Department and Neurology Service, University Hospitals of Strasbourg, 67000 Strasbourg, France; CM2R (Research and Resources Memory Centre), Geriatric Day Hospital and Neuropsychology Unit, Geriatrics Department and Neurology Service, University Hospitals of Strasbourg, 67000 Strasbourg, France; ICube Laboratory UMR 7357 and FMTS (Fédération de Médecine Translationnelle de Strasbourg), IMIS Team and IRIS Platform, University of Strasbourg and CNRS, 67000 Strasbourg, France; CM2R (Research and Resources Memory Centre), Geriatric Day Hospital and Neuropsychology Unit, Geriatrics Department and Neurology Service, University Hospitals of Strasbourg, 67000 Strasbourg, France; ICube Laboratory UMR 7357 and FMTS (Fédération de Médecine Translationnelle de Strasbourg), IMIS Team and IRIS Platform, University of Strasbourg and CNRS, 67000 Strasbourg, France; ICube Laboratory UMR 7357 and FMTS (Fédération de Médecine Translationnelle de Strasbourg), IMIS Team and IRIS Platform, University of Strasbourg and CNRS, 67000 Strasbourg, France; CM2R (Research and Resources Memory Centre), Geriatric Day Hospital and Neuropsychology Unit, Geriatrics Department and Neurology Service, University Hospitals of Strasbourg, 67000 Strasbourg, France

**Keywords:** autobiographical memory, Autobiographical Interview self, insula, dementia with Lewy bodies

## Abstract

Although deficits in learning and retrieving new information are well characterized in dementia with Lewy bodies, autobiographical memory has never been explored in this disease. Yet, autobiographical memory impairments are a pervasive feature of dementia, well characterized in other neurodegenerative diseases. Moreover, autobiographical memory corresponds to an extension over time of the self, which we hypothesize is altered in dementia with Lewy bodies and impairment of which could be linked to the insular atrophy occurring from an early stage of the disease. In this study, we sought to characterize autobiographical memory impairments and explore their neural correlates in dementia with Lewy bodies, on the assumption that insular damage could impact the self, including its most elaborate components, such as autobiographical memory. Twenty patients with prodromal to mild dementia with Lewy bodies were selected to participate in this exploratory study along with 20 healthy control subjects. The Autobiographical Interview was used to assess autobiographical memory. Performances were compared between patients and control subjects, and an analysis across life periods and recall conditions was performed. 3D magnetic resonance images were acquired for all participants, and correlational analyses were performed in the patient group using voxel-based morphometry. The behavioural results of the Autobiographical Interview showed that autobiographical memory performances were significantly impaired in dementia with Lewy body patients compared to control subjects in a temporally ungraded manner, for both the free recall and the specific probe conditions (*P* < 0.0001), though with greater improvement after probing in the patient group. Furthermore, autobiographical memory impairments were correlated with grey matter volume within right insular cortex, temporoparietal junction, precuneus, putamen, left temporal cortex, bilateral parahippocampus and cerebellum, using a threshold of *P* = 0.005 uncorrected. The behavioural results confirm the existence of temporally ungraded autobiographical memory impairments in dementia with Lewy bodies, from the early stage of the disease. As we expected, neuroimaging analysis revealed a role for the insula and the precuneus in autobiographical memory retrieval, two regions associated with elementary aspects of the self, among other brain regions classically associated with autobiographical memory, such as medial temporal lobe and temporoparietal junction. Our findings provide important insights regarding the involvement of the insula in the self and suggest that insular damage could lead to a global collapse of the self, including its more elaborated components, such as autobiographical memory.

## Teaser

Tisserand *et al*. report that patients with prodromal to mild dementia with Lewy bodies display autobiographical memory impairments, with a flat profile across life epochs. Moreover, they found that these impairments would be partly related to insular atrophy that occurs early in the disease.

## Graphical Abstract

**Graphical Abstract:**
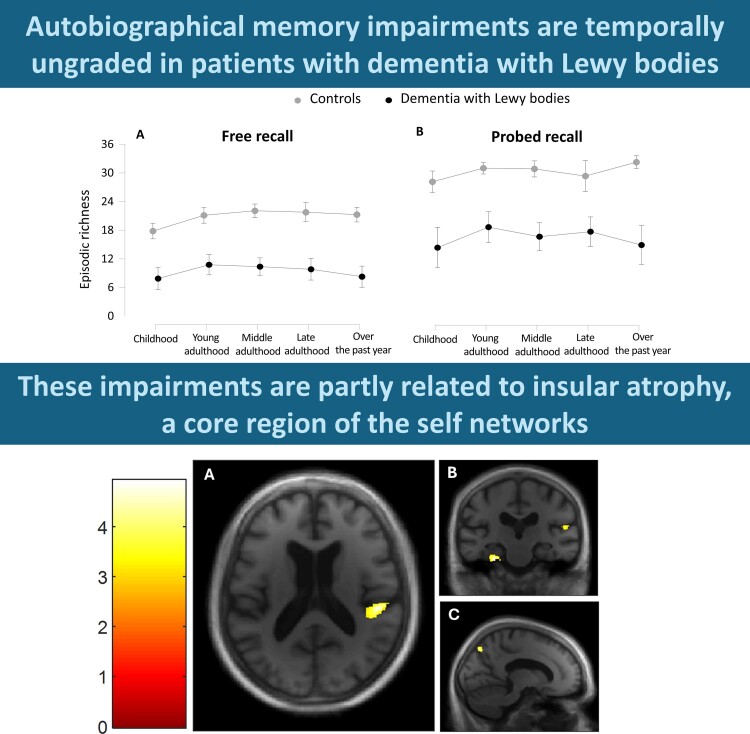

## Introduction

While pervasive deficits in learning and retrieving new information are well characterized in dementia with Lewy bodies,^1,2^ autobiographical memory (ABM) has never been explored in this disease. Yet, in dementia with Lewy bodies, there seem to be obvious alterations of the present self,^3-7^ which has been proposed as a prerequisite for the self extended in time, namely ABM.^8-10^ The present self pertains to the aspects of the sense of self that are related to and accessible in the present moment. According to Prebble *et al*.,^9^ the present self comprises two components: the subjective sense of self and the self-concept. The subjective sense of self refers to the subjective living experience that implies the construction of a mental representation,^11-13^ whereas the self-concept contains the object of this representation, including knowledge about ourselves.^12,14^ Recently, a study in dementia with Lewy body patients has shown deficits in awareness, such as anosognosia^3^ that could reflect alteration of the subjective sense of self, while other studies demonstrated that dementia with Lewy body patients display changes in their personal tastes and impairments in representing who they are,^5,15^ revealing alteration of the self-concept. In these latter studies, we were able to link the alteration of the self to the insula, which is damaged early in the course of dementia with Lewy bodies^16-19^ and is a core region for the self.^15^ Indeed, the insula is involved in multiple processes related to the present self, ranging from internal bodily states, such as interoception,^20,21^ to high-order processes, such as knowledge about oneself,^7,22^ but little is known about its contribution to the self extended in time.

ABM refers to the capacity to recollect experiences personally lived and to autobiographical knowledge, supporting the construction of the representation of our identity over time.^23^ ABM is a complex construct that can be dissected into two distinct memory systems: an episodic one and a semantic one.^10,24^ The episodic component refers to the subjective re-experiencing of events from the past, with emotions and perceptual details, within a particular place and time, whereas the semantic components consist of knowledge, facts and conceptual information related to personal life, devoid of the spatio-temporal context in which this information was acquired.^25^ However, an obvious interconnection between the self and ABM is recognized by expert researchers in the field.^8,9,21,26,27^ A growing number of studies have investigated the neurobiological basis of this complex form of cognition. In a meta-analysis conducted using haemodynamic imaging in healthy adults,^28^ researchers identified a core ABM network, comprising medial temporal lobe structures, including the hippocampus and parahippocampal cortices, the lateral temporal cortices, the medial prefrontal, posterior parietal region and the cerebellum, whereas the lateral prefrontal and temporal cortices, insula, precuneus and basal ganglia appeared less frequently involved. To our knowledge, only two studies found a correlation between the insula and ABM. In the first study, Fink *et al*.^29^ found the insula to be a secondary region of ABM in a healthy population. In the second study, Descamps *et al*.^30^ found a diminished ABM richness in patients with insular resection compared to healthy controls. Moreover, some behavioural studies found impaired ABM in disorders associated with insular dysfunction, such as schizophrenia and autism spectrum disorders,^31,32^ though no imaging study to confirm potential insular involvement was included.

Impaired ability to remember events from the past is considered a transdiagnostic feature of dementia and is often among the earliest symptoms reported by patients. ABM impairments can take different forms arising from the dysfunction of neurocognitive processes, depending on the distribution of neural atrophy in the brain.^33^ In Alzheimer’s disease, episodic memory dysfunction caused by neuronal degeneration in the medial temporal lobe leads to an extended retrieval deficit due to a global degradation of specific ABM,^34,35^ more pronounced for the recent period due to an additional encoding/consolidation deficit for recent memories.^36,37^ Meanwhile, semanticized memories from remote periods of life are better preserved given that they rely on the relative integrity of the temporal neocortex.^38^ Conversely, semantic dementia is characterized by damage to the anterior temporal neocortex^39^ and by a ‘reversed’ ABM profile, with spared recent episodic memories^40^ contrasting with the loss of remote semanticized memories.^35,41^ In the behavioural variant of frontotemporal dementia, which is characterized by damage to the frontoinsular and ventromedial prefrontal cortices,^42,43^ the ABM profile appears flat due to multiple mechanisms, including strategic retrieval difficulties.^33,35,40,44^ A similar ABM flat profile is described in posterior cortical atrophy and would be related to disruption of access to the visual contextual information integral to the ABM trace, in connection with atrophy of posterior parietal regions, including the right précuneus.^45^

Despite the evidence of altered ABM in the aforementioned dementia syndromes, no studies have yet explored this type of memory in dementia with Lewy bodies, which is, however, the second most common form of cognitive neurodegenerative disease after Alzheimer's disease.^46,47^ Thus, in this paper, we report the first behavioural and neuroimaging study of ABM in dementia with Lewy body patients. Our purpose was to explore the potential ABM deficit in dementia with Lewy bodies along with its anatomical substrates, with a particular interest in the insula. Based on the premise that the insula is damaged at an early stage in dementia with Lewy body patients and based on the assumption that the insula plays a central role in the self, we predicted that ABM would be impaired in an ungraded manner due to a global alteration of the self. Moreover, given that memory disorders in dementia with Lewy bodies are characterized by deficits in learning and retrieving the information, we expected that retrieval support would have beneficial effects on the richness of memories.

## Materials and methods

### Study population

The current study was conducted using the same cohort of patients as in our previous research on the self.^6^ Specifically, it included 20 patients with early-stage dementia with Lewy bodies and 20 healthy control subjects matched for age, gender and level of education (with a minimum of 9 years) were enrolled in the present study between January 2021 and February 2023. Patients were recruited from the tertiary memory clinic of Strasbourg University Hospital (CM2R of Strasbourg), France, including the geriatrics and neurology departments. Control subjects were recruited in three ways: among friends and relatives of the patients, via the listing of controls of the local clinical investigation centre and from the control group of the AlphaLewyMA cohort (http://clinicaltrials.gov/ct2/show/NCT01876459). Diagnosis of prodromal and mild dementia with Lewy bodies was based on core clinical features.^48,49^ Some of the patients had also benefited from biomarkers during their clinical follow-up. Indeed, a dopamine transporter (DAT) imaging was performed when parkinsonism was doubtful, and a CSF analysis was performed when an amnestic syndrome of hippocampal type suggested possible Alzheimer’s disease, to ensure that there was no co-pathology. Thus, a DAT scan was available to support the diagnosis in approximately a quarter of the patients, and CSF analysis was available to confirm the absence of associated Alzheimer’s disease in approximately half of the patients. Patients with prodromal dementia with Lewy bodies were defined as having mild cognitive impairment if they had a Mini-Mental State Examination (MMSE) score of ≥26, had preservation of independence as assessed by the Instrumental Activities of Daily Living^50^ and fulfilled both the DSM-5 criteria of mild neurocognitive disorder^51^ and McKeith’s criteria for the diagnosis of prodromal dementia with Lewy bodies.^48^ Patients were defined as having mild dementia with Lewy bodies if they had an MMSE score between 20 and 25 and were diagnosed as having probable dementia with Lewy bodies according to the current dementia with Lewy body criteria.^49^ All participants benefited from a classic medical examination, which notably included evaluation of the features of parkinsonism using the Unified Parkinson’s Disease Rating Scale (Part 3): akinesia, rigidity and resting tremor (rated from 0 for no symptoms to 4 for serious symptoms). The dementia with Lewy bodies group underwent further clinical examination of dementia with Lewy body core criteria, among which fluctuations were assessed with the Mayo Clinic Fluctuations Scale^52^ and the Newcastle-upon-Tyne Clinician Assessment of Fluctuation Scale.^53^ The Parkinson’s disease-associated psychotic symptoms questionnaire^54^ was used to evaluate the presence of hallucinations. Rapid eye movement sleep behaviour disorder (RBD) was evaluated using a sleep questionnaire on RBD,^55^ simplified into four questions, two each for the patient and for the caregiver: one concerning movements during sleep and the second concerning vivid dreams and nightmares.

Subjects with a history of alcohol/substance abuse, significant visual or auditory disabilities, relevant neurological of psychiatric comorbidities or the presence of other severe or unstable medical illnesses were not enrolled in the study. Subjects with an abnormal neurological examination—except for parkinsonism in the case of patients—depression symptoms (mini-Geriatric Depression Scale^56^) or a significant cerebral vascular burden (Modified Hachinski Ischemic Score of >7^57^) were not enrolled. Participants with CSF biomarkers suggestive of Alzheimer’s disease (i.e. abnormal Aβ42/Aβ40 ratio, t-Tau, phospho-Tau181) were not enrolled. Finally, participants with claustrophobia or contraindications to MRI were not enrolled. All participants provided written informed consent for the study, in accordance with the Declaration of Helsinki, and the study was approved by the ethics committee Sud Méditerranée III.

### Behavioural study

#### Baseline

General cognitive assessment was performed in the patients, including an evaluation of anterograde memory with the RL/RI-16 (‘Rappel libre/Rappel indicé à 16 items’, i.e. 16-item free recall/probed recall),^58^ a French test similar to the FCSRT (Free and Cued Selective Reminding Test),^59^ verbal fluencies,^60^ Frontal Assessment Battery,^61^ TMTA and TMTB,^62^ digit span of the WAIS (Wechsler Adult Intelligence Scale)^63^ and Rey-Osterrieth Complex Figure.^64^ The results are displayed in Supplementary Table 1.

#### Autobiographical Interview

The Autobiographical Interview^25^ was administered and rated according to Levine’s methodology,^65^ for an analysis of the participants ability to relate, under different levels of retrieval support, autobiographical memories established at different times in their lives. Participants were instructed to provide detailed descriptions of two events that were personally experienced and that occurred at a specific time and place from each of five life periods (childhood, age 0–15 years; young adulthood, age 16–30; middle adulthood, age 31–50; late adulthood, age 51–the past year; during the past year). In the original version of the Autobiographical Interview,^25^ only one event per life period was required, but in the present study, participants were asked to describe two events, so as to increase the quantity of material to be analysed. Life periods were randomly assigned during the session, and a list of 20 word cues (e.g. car, piano, apple) was presented to assist in event retrieval.

To examine facilitative effects of retrieval support on memory, we manipulated the level of structure available to participants across three conditions: free recall, general probe and specific probe. At free recall, participants spoke about the event without any interruption from the examiner, continuing until they had reached a natural ending point. After an event had been recalled, the examiner prompted the participant once for a greater recall of details (general probe). At the specific probe phase, a structured interview was administered to elicit additional contextual details encompassing four categories: time, place, perceptual details and emotion/thought.

Descriptions were recorded and transcribed. Following administration, event descriptions were segmented into details and classified as ‘internal’ and assigned to one of five categories (event, place, time, perceptual and emotion/thought) if they related directly to the main event described, were specific to time and place and conveyed a sense of episodic re-experiencing. Otherwise, details were considered ‘external’, consisting of satellite autobiographical events or unrelated to the main event, semantic facts, repetitions or other metacognitive statements. Details from each category were summed separately for each condition (i.e. free recall, general probe, specific probe). As the effect of general probing on general performance is minimal in comparison to that of specific probing,^25^ free recall and general probe scores were combined. Quantitative ratings were accompanied by qualitative ratings assigned for time, place, perception and emotion/thought, with the possibility of attaining a maximum rating of 3 points for each category (time, place, perceptual, emotion/thought). A rating of 3 points was assigned when the description was rich, highly specific and appeared to emerge from a feeling of re-experiencing. A rating of 2 points was assigned to detailed descriptions falling short of a 3-point description. A rating of 1 point was assigned to descriptions containing general, non-specific information but still episodic in nature. A rating of 0 point was assigned when there was no information pertaining to the specified category, or for responses based on semantic knowledge rather than episodic memory. Episodic richness (the overall degree to which a feeling of re-experiencing was conveyed) was rated on a similar scale that was extended to 6 points to provide a more accurate rating and to take into account the greater importance of this category relative to the others. Although ratings in the first four categories were mutually exclusive (i.e. aspects of a memory could not be counted in more than one category), the episodic richness was based on an overall assessment of the event. To investigate potential differences across time periods, the episodic richness ratings of the two memories were added for each period for both the free recall (i.e. free recall plus general probe) and the probed recall (i.e. total score following probing) (maximum = 36). To analyse the possible differences between the two recall conditions, the scores of each period were added up to obtain a general free recall score and probed recall score (maximum = 180).

Participants’ memories were scored by one trained rater (A.T.). To assess interrater reliability, 10% of the memories were selected at random (with the constraint that age, groups and life periods were equally represented) and scored by another trained rater (N.P.) who was blind to subject status, in accordance with established scoring procedures from previous research.^25,66^ Interrater reliability was high for free recall score and probed recall score (all Cronbach’s *α* ≥ 0.94).

### Statistical analysis for the behavioural study

Student’s *t*-tests were used to compare intergroup differences between dementia with Lewy body patients and control subjects for demographic quantitative data. A *χ*^2^ test was used to compare the sex ratio between groups. For the behavioural data, Mann–Whitney U-tests were used to compare the free recall score and probed recall score,^25^ for both dementia with Lewy body patients and control subjects, as we hypothesized that scores would be lower in the free recall condition compared to the specific probe. We also compared the percentage of improvement after specific probe ((probed recall score-free recall score/free recall score) × 100) between dementia with Lewy body patients and control subjects, using Mann–Whitney U-tests. Two-way repeated measures ANOVAs, including age as nuisance covariate, were conducted to examine the effect of life period across the different recall conditions, for both dementia with Lewy body patients and control subjects. Bonferroni correction was applied for multiple comparisons.

### Neuroimaging study

As described previously,^6^ each participant underwent a high-resolution anatomical MRI scan within a maximum of 12 weeks after taking the Autobiographical Interview. T_1_-weighted 3D anatomical images were obtained using a 3T MRI scanner (Verio 32-channel Tim Siemens scanner; Siemens, Erlangen, Germany) using a volumetric magnetization-prepared rapid acquisition with gradient-echo (MPRAGE) sequence (field of view = 256 × 256 mm, image matrix = 256 × 256, slice thickness = 1 mm, repetition time = 1900 ms, echo time = 2.52 ms, flip angle = 9°).

### Voxel-based morphometry analyses

We used voxel-based morphometry (VBM) to investigate differences in grey matter (GM) volume between the healthy controls and the dementia with Lewy body patients and to examine the neuroanatomical correlates of ABM in dementia with Lewy body patients. VBM analyses included image preprocessing and statistical analyses. These steps were carried out using the SPM12 software package (Wellcome Department of Imaging Neuroscience, London; http://www.fil.ion.ucl.ac.uk/) running on Matlab R2017b (MathWorks, Natick, MA, USA). Anatomical MRI images were spatially preprocessed using standard procedures.^67^ All T_1_-weighted structural images were first segmented, bias corrected and spatially normalized to the Montreal Neurological Institute space using an extension of the unified segmentation procedure that includes six classes of tissue.^68^ The DARTEL registration toolbox was then used to build a study-specific template and to bring into alignment all of the segmentation images. The VBM analysis was done on modulated GM images; that is, the GM value in each voxel was multiplied by the Jacobian determinant derived from the spatial normalization. This procedure preserves the total amount of GM from the original images. These modulated GM images were smoothed with a Gaussian kernel (full width at half maximum: 8 mm). Between-group voxel-based comparisons were displayed after correcting for multiple comparisons with false discovery rate (FDR; *P* < 0.05). A voxel-wise general linear model was employed to investigate the regions of atrophy related to disorders of the episodic ABM, in dementia with Lewy body patients. To analyse the potential different regions involved in the free recall and in the probed recall, we tested the correlation between the GM volume and both the free recall score and the probed recall score, using a threshold of *P* = 0.005 uncorrected, including age, gender and total intracranial volume (TIV) as nuisance covariates. MMSE was also considered as an additional covariate to investigate the potential impact of disease severity. Moreover, to obtain a control measure of memory not involving the self, we tested the correlations between GM volume and the 1–3 free and total recall conditions of the RL/RI-16,^58^ using the same threshold, including the same nuisance covariates as for the Autobiographical Interview. A cluster spatial extent of 50 voxels was used to avoid irrelevant and isolated detections. The software xjView (http://www.alivelearn.net/xjview8/) enabled us to characterize each cluster.

## Results

### Clinical characteristics

Twenty patients with early-stage dementia with Lewy bodies and 20 healthy control subjects were enrolled in the present study. A description of the study population is presented in Table 1. Groups were well matched for age (*t* = 0.64, *P* = 0.526), years of education (*t* = −1.61, *P* = 0.116) and sex (*χ*^2^ = 0.44, *P* = 0.507). The dementia with Lewy bodies group comprised 13 patients at the prodromal stage of dementia with Lewy bodies and 7 patients at the mild stage of the disease. Among the 20 dementia with Lewy body patients, 85% presented fluctuations, 60% presented hallucinations or illusions and 60% presented RBD. Concerning parkinsonism features, akinesia was observed in 80% of dementia with Lewy body patients, rigidity in 70% of dementia with Lewy body patients and resting tremor in only one patient (5%).

**Table 1:** Demographic and clinical characteristics of the dementia with Lewy bodies group and the control group

Characteristic	Dementia with Lewy bodies group (*n* = 20)	Control group (*n* = 20)	Student’s *t*-test or *χ*^2^ test
Mean age in years (+SD)	71.9 (7.9)	70.4 (7.3)	*t* = 0.64, *P* = 0.526
Years of education (+SD)	12.7 (2.5)	14.05 (2.6)	*t* = *−*1.61, *P* = 0.116
Sex, M/F	14/6	12/8	*χ^2^* = 0.44, *P* = 0.507
MMSE score (/30)	26.3 (2.39)	28.8 (1.0)	*t* = *−4.32*, *P* < 0.001
Handedness, R/L	19/1	20/0	χ*^2^* = 1.03, *P* = 0.311
Mean of disease duration (+SD)	3.19 (2.12)	N/A	
Fluctuations (%)	17/20 (85)	N/A	
Hallucinations/illusions (%)	12/20 (60)	N/A	
RBD (%)	12/20 (60)	N/A	
Parkinsonism			
Akinesia (%)	16/20 (80)	N/A	
Rigidity (%)	14/20 (70)	N/A	
Tremor at rest (%)	1/20 (5)	N/A	

Significant *P*-value and *χ*^2^ value are in italics. SD, standard deviation; M, male; F, female; MMSE, Mini-Mental State Examination; R, right; L, left; RBD, rapid eye movement sleep behaviour disorder; N/A, not applicable.

### Behavioural study

#### Overall performance on the Autobiographical Interview

**Figure 1:**
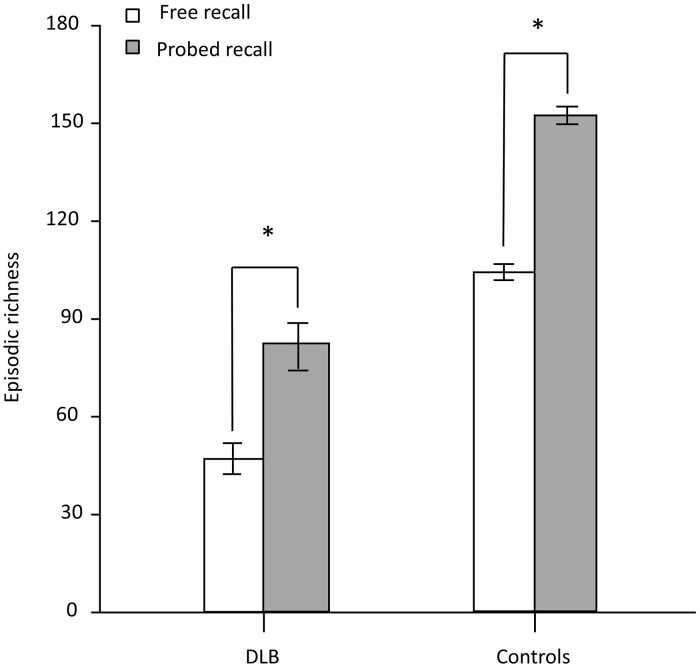
**Total scores of episodic richness for the free recall and probed recall on the Autobiographical Interview, for all participants.** Mann–Whitney U-tests were used to compare the free recall score and probed recall score for both dementia with Lewy body patients (*n* = 20) and control subjects (*n* = 20). *P < 0.0001 (error bars represent standard deviation). DLB, dementia with Lewy bodies.

Figure 1 shows the total scores of episodic richness summed across all time periods in the free and probed retrieval conditions of the Autobiographical Interview. Mann–Whitney U-test revealed that controls displayed higher episodic richness across both retrieval conditions in comparison to dementia with Lewy body patients (*P* < 0.0001). The mean score obtained in free recall was significantly inferior in the dementia with Lewy bodies group (46.9, SD 21.02) than in the control group (103.95 SD 11.17) (*U* = 2.5, *P* < 0.0001). Similarly, the mean score obtained in probed recall was significantly inferior in the dementia with Lewy bodies group (82.3, SD 31.68) than in the control group (152.1, SD 12.36) (*U* = 2.5, *P* < 0.0001). The episodic richness evolution rate between the free recall score and the probed recall score was higher in dementia with Lewy body patients (gain of 75.48% of episodic richness in the probed recall) compared to controls (gain of 46.32% of episodic richness in the probed recall) (*U* = 341, *P* < 0.0001).

#### ABM retrieval across life periods

**Figure 2:**
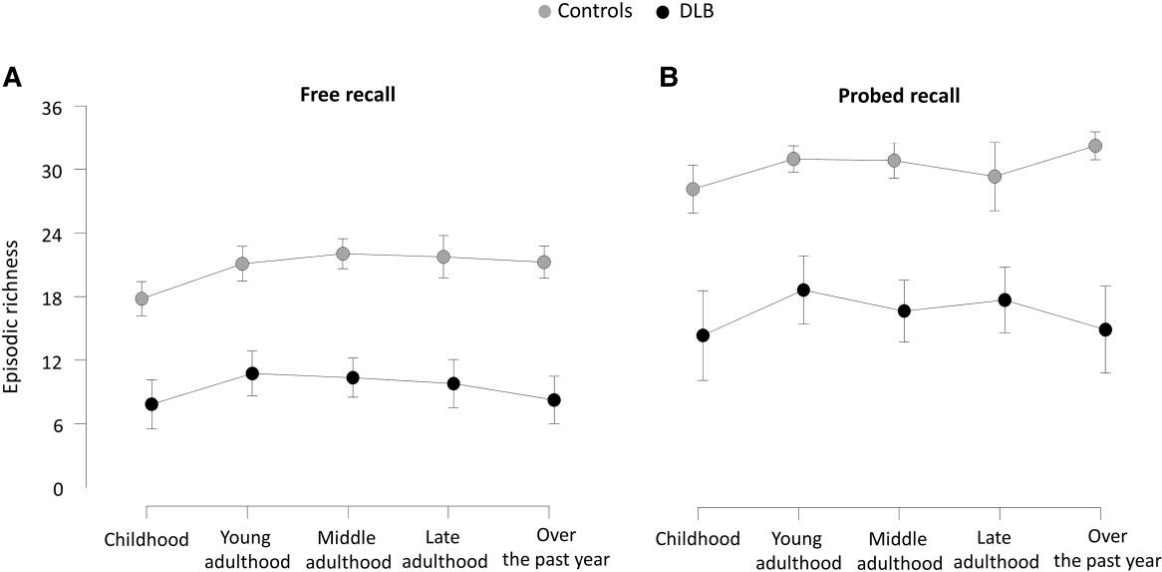
**Profile of free recall (A) and probed recall (B) across life periods on the Autobiographical Interview for all participants.** Two-way repeated measures ANOVAs, including age as nuisance covariate, were conducted to examine the effect of life period across the different recall conditions, for both dementia with Lewy body patients (*n* = 20) and control subjects (*n* = 20). Bonferroni correction was applied for multiple comparisons (error bars represent standard deviation). DLB, dementia with Lewy bodies.

Figure 2 shows the profile of free recall and probed recall across life periods. A two-way repeated measures ANOVA revealed significant main effect for group [*F*(1,37) = 98.93, *P* < 0.0001], with controls displaying overall higher episodic richness than dementia with Lewy body patients (*t* = 9.95, *P* < 0.001). Another significant main effect was found for recall condition [*F*(1,37) = 9.83, *P* = 0.0034], with significantly higher episodic richness in the probed recall condition for both dementia with Lewy body patients and controls (*t* = −23.23, *P* < 0.001). There was also a significant interaction between group and recall condition [*F*(1,37) = 11.16, *P* = 0.0019], with significantly lower scores in the dementia with Lewy bodies group compared to the control group for both free recall (*t* = 8.62, *P* < 0.001) and probed recall (*t* = 10.48, *P* < 0.001) conditions. No significant main effect was found for life periods for both controls and dementia with Lewy body patients, in either the free recall condition or the probed recall condition. Other comparisons did not reveal significant differences between the scores.

### Neuroimaging study

A voxel-based analysis comparing GM volume in dementia with Lewy body patients versus control subjects is presented in Supplementary Fig. 1. The analysis included TIV and age as nuisance covariates and revealed patterns of cerebral atrophy typically reported in dementia with Lewy body patients,^17,69^ with reduction in GM volume in the insular, temporal, occipital, frontal and cingular cortices, and to a lesser extent the parietal cortex, and also in the cerebellum and subcortical regions such as the putamen (*P* < 0.05, FDR corrected), when compared to control subjects.

**Figure 3:**
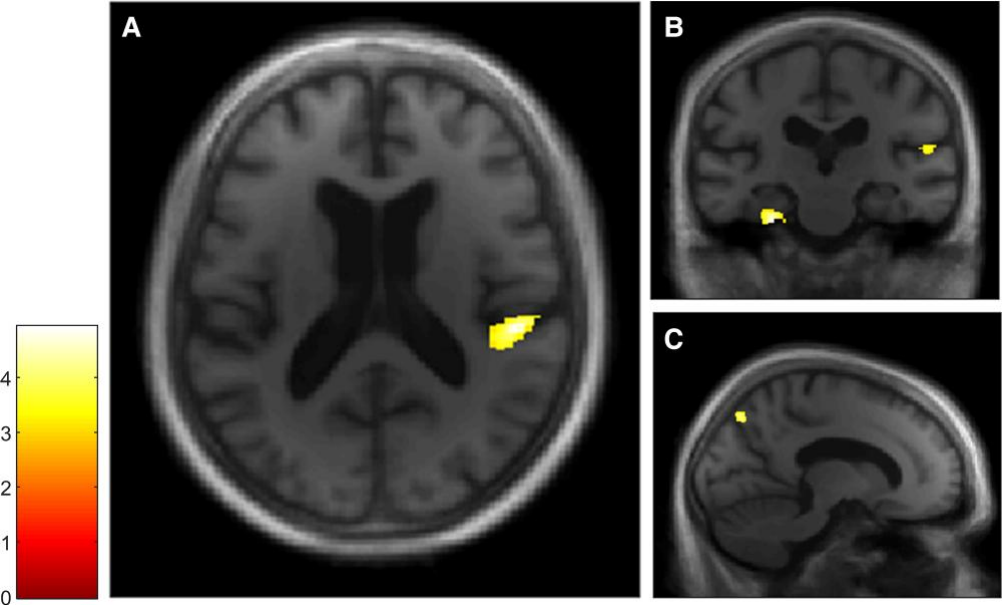
**VBM analyses for the Autobiographical Interview free recall condition in the dementia with Lewy bodies group.** Multiple linear regression was used to obtain correlation for Autobiographical Interview free recall score and GM volume in patients with dementia with Lewy bodies (*n* = 20). GM volumes within right posterior insula (**A** and **B**), left parahippocampal gyrus and right temporoparietal junction (**B**) and right precuneus (**C**) are positively correlated with the free recall score on the Autobiographical Interview, using a threshold of *P* = 0.005 uncorrected, including age, gender, TIV and MMSE score as nuisance covariates (*k* = 50).

**Figure 4:**
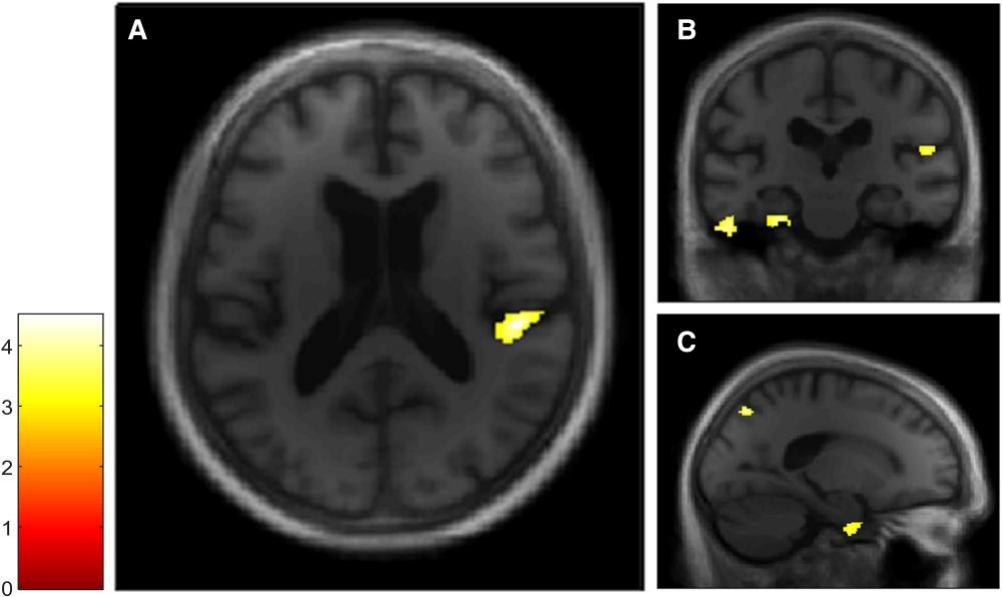
**VBM analyses for the Autobiographical Interview probed recall condition in the dementia with Lewy bodies group.** Multiple linear regression was used to obtain correlation for Autobiographical Interview probed recall score and GM volume in patients with dementia with Lewy bodies (*n* = 20). GM volumes within right posterior insula (**A** and **B**), left inferior temporal and parahippocampal gyri (**B**) and right precuneus and parahippocampal gyrus (**C**) are positively correlated with the probed recall score on the Autobiographical Interview, using a threshold of *P* = 0.005 uncorrected, including age, gender, TIV and MMSE score as nuisance covariates (*k* = 50).

The free recall score and the probed recall score were analysed separately to examine facilitative effects of retrieval support on memory. VBM analyses revealed a positive correlation between both the free recall score (Fig. 3) and the probed recall score (Fig. 4) and GM volumes within a total of seven clusters, four of which were common to both Autobiographical Interview conditions (see Table 2). The first cluster included the right posterior insular cortex and the temporoparietal junction, the second cluster included the right precuneus, the third cluster included the right parahippocampal gyrus and the fourth cluster included the left parahippocampal gyrus and the left cerebellum. In the free recall condition, other clusters were found in the right cerebellum and in the right putamen, whereas in the probed recall condition, another cluster was found in the left inferior temporal gyrus.

**Table 2:** VBM results for the free recall and probed recall conditions on the Autobiographical Interview in the dementia with Lewy bodies group

				*K*	
Cluster	VBM	Side	BA	FRS	PRS
1.	Posterior insular cortex	R	13	216/813	226/765
	Temporoparietal junction	R	41	361/813	305/765
2.	Precuneus	R	7	97/112	184/194
3.	Parahippocampal gyrus	R	28/36	120/126	509/544
4.	Parahippocampal gyrus	L	35/36	393/701	423/709
	Cerebellum	L	NA	109/701	104/709
5.	Cerebellum	R	NA	93/100	
6.	Putamen	R	NA	92/195	
7.	Inferior temporal gyrus	L	20		77/137

Please note that the entire table with exact Talairach coordinates is provided in Supplementary Table 2. FRS, free recall score; PRS, probed recall score; L, left; R, right; BA, Brodmann area; *K*, cluster size in voxels (specific region’s volume/cluster’s global volume).

VBM analyses for both the 1–3 free and total recall at the RL/RI-16 did not reveal any association with the insula. The results are presented in Supplementary Figs 2 and 3.

## Discussion

This study explored the anatomical correlates and the profile of episodic ABM impairments in patients with early-stage dementia with Lewy bodies. The main aim was to determine whether the insula, as a core region of the self,^15^ is involved in the self extended in time in dementia with Lewy body patients. In summary, in the behavioural study, our findings confirm that ABM is impaired in patients with early-stage dementia with Lewy bodies, in an ungraded manner impacting all time periods, partially linked to strategic retrieval deficits, but not exclusively. Neuroimaging analyses confirm that the insular cortex is associated with ABM, namely in the self extended in time, aside from its well-known role in the present self. Additionally, we found that another crucial region of the self, the precuneus, is also associated with ABM. Other well-known regions of ABM retrieval networks, such as the parahippocampal gyri and the parietotemporal junction, were also correlated with ABM richness. Finally, the cerebellum and the putamen were correlated with ABM free recall, whereas the temporal lobe was only correlated with ABM probed recall. These results bring new insights into the role of the insular cortex in ABM, into the reliance of ABM on more elementary aspects of the self and into the behavioural characteristics of dementia with Lewy body patients.

Empirical evidence suggests that ABM impairments are a hallmark feature of dementia, such as in Alzheimer’s disease, as well as in other dementia syndromes such as semantic dementia and frontotemporal lobar degeneration.^33,44^ However, our study is the first to demonstrate that ABM is impaired in dementia with Lewy bodies. The present findings show an improvement in the episodic richness of memories through the provision of structured probing, notably for the dementia with Lewy body patients who displayed higher evolution rate of episodic richness, which grew by 75% in the probed recall versus the free recall, while the controls grew by 46%. These results reinforce the idea that ABM impairments could partially pertain to a failure of the strategic retrieval process, due to the executive dysfunction characterizing dementia with Lewy bodies.^2^ However, despite the provision of structured probing, patients continued to display reduced episodic re-experiencing when compared to control subjects, suggesting inability to access previously stored memories entirely. Importantly, we found that ABM impairments were temporally ungraded in dementia with Lewy body patients, with a flat performance of episodic re-experiencing across life epochs, including performance for the recent period contrary to what has been documented in Alzheimer's disease or in semantic dementia. Indeed, the neuroanatomical signature of Alzheimer's disease affects the encoding of new information and the storage of the episodic memory trace, which affects the recent period to a greater extent,^36,37^ whereas recent episodic memories are relatively spared in semantic dementia.^40^

In dementia with Lewy bodies, this finding of a flat retrieval profile without greater impairment for the recent period could be related to a breakdown in research strategies,^33^ whose probing would allow access to stored memories up to a limit. This flat profile could also be partially explained by a storage deficit; however, we would expect to find greater impairment for the recent period if such a deficit were to be at the forefront. Alternatively, as ABM and the subjective experience are closely linked,^9^ another hypothesis could be that the subjective sense of self might be impaired, thus leading to alteration of autonoetic consciousness and disturbing access to memories, regardless of the period,^9,70^ as it has already highlighted in pathology with flat profile of ABM.^35^ This postulate could be reinforced by the correlation between ABM and insular atrophy that is the central finding to emerge from our study.

Indeed, concordant with our hypothesis, imaging analyses confirmed that the insular cortex, and more precisely its right posterior part, is specifically associated with ABM, whereas it was not involved in anterograde verbal memory on the RL/RI-16, which performance reflects general memory processes. The insular cortex is commonly reported to be associated with interoceptive awareness^71^ and socio-emotional processes,^21^ but also with social recognition memory,^72^ taste memory^73^ and recognition memory formation and consolidation.^74-76^ Regarding dementia with Lewy bodies, our study is the first to suggest an impoverished ABM in relation to the insular atrophy occurring early in the course of the disease,^16-19^ whereas the insula has been mentioned in only a few studies on ABM and is often considered as a region of secondary importance. Yet, the role of the insula is already well known in both the subjective and objective components of the present self.^5,7,20-22,77-79^ As mentioned above, the subjective sense of self can readily be assessed by measuring interoceptive awareness, sense of body ownership and sense of agency and metacognition. Yet, numerous studies found that these different levels of the subjective sense of self are indeed associated with the right insula.^80-83^ Specifically, the most elementary aspects of the subjective sense of self, namely interoception, sense of agency and sense of body ownership, involve the posterior insular cortex.^71,84-88^ The self-concept, however, encompasses a body of autobiographical knowledge, self-esteem and self-image and has also been related to the insula in several studies.^22,83,89^ Our team recently highlighted a correlation between poorer knowledge about oneself and diminished GM volume in the right insula in dementia with Lewy bodies.^6^ The subjective sense of self and the self-concept are respectively elementary preconditions for episodic and semantic ABM,^10^ which in turn is central for the formation and maintenance of identity in the present moment and a continuous mental representation of the self over time.^8-10,24,27,90,91^ Thus, our findings suggest that the insula is a key region of the network of the self extended in time, its atrophy leading to an ABM deficit, likely through an alteration of the self at the present moment. Moreover, the fact that the posterior part of the insular cortex is involved suggests that the most elementary aspects of the self could be involved in ABM.^71,84-88^ This in in line with our hypothesis whereby alteration of the most elementary component of the self, namely the subjective sense of self, would lead to impairments of higher order self-components such as ABM. However, the insula is not commonly found as a core region in other studies on ABM. An explanation could be that ABM studies in healthy subjects show a prominent involvement of brain regions involved in more elaborated aspects of the self, as in the case of the cortical midline structures, in addition to regions involved in episodic recollection. In dementia with Lewy bodies, we can speculate that it is the insular damage that prevents the grounding of the networks of the self, such as the ABM network, and leads to a global collapse of the self. Conversely, when the insula and the subjective sense of self are preserved, we can surmise that their activation is in the background, compared to activation in regions involved in episodic recollection and in more elaborated aspects of the self.

Our second main finding concerns the relationship between the right precuneus and autobiographical memories. The precuneus is considered part of the cortical midline structures,^92^ which are a complex of brain regions involved in the self networks. Thus, it is already known to be a crucial component of the self, notably for processing of bodily self, namely the subjective sense of self,^93-95^ but also in self-referential processing, namely the self-concept.^83,93,96-99^ Beyond its role in the present self, the precuneus has also been reported to have a role in ABM, though not as a core region.^28^ Interestingly, some researchers found that the precuneus was specifically involved in the retrieving of specific autobiographical events, as opposed to general past memories^100^ and personal semantic information,^101^ which is consistent with the notion of the precuneus being more likely related to the self and personal aspects, rather than directly to the memory aspects. Moreover, the precuneus is involved in visuo-spatial processes^102^ and visual imagery processes occurring in conscious memory recall.^103,104^ Our results in dementia with Lewy body patients are in line with a study on patients with posterior cortical atrophy^45^ who displayed a disruption of visual and perceptual processing, as reported in dementia with Lewy body patients.^2^ Ahmed *et al*. found a specific correlation between perceptual details in ABM and GM density in the precuneus; thus, impairments in perception processing and the deprivation of visual imagery could impact recollection in ABM, acting like a barrier to mental travel in memories. Furthermore, another finding of our study, which has extensively been explored and associated with specific autobiographical memories, is the temporoparietal junction,^28^ which is also known to be specifically involved in retrieval of the spatial context of events.^105^

In line with previous studies exploring ABM, we found an involvement of the medial temporal lobe, which is part of the ‘core network’ of ABM^28^ and has already been described in healthy subjects^25,106^ and in Alzheimer's disease.^37,107^ Indeed, aside from its well-known role in the formation and consolidation of newly learned material,^106,108,109^ the hippocampus plays a central role in autobiographical recollection, serving as an index pointing to details of the memories stored in parts of the neocortex.^28^ Nevertheless, our study found no involvement of the hippocampus, but rather an involvement of bilateral parahippocampal structures, with clusters also encompassing areas of the perirhinal and entorhinal cortices, namely Brodmann area (BA) 35/36 and BA 28. This is likely due to the fact that dementia with Lewy bodies would relatively spare CA1, the larger subregion of the hippocampus, whereas neuropathological lesions are rather found in the more restricted CA2 subfield and the entorhinal cortex.^110^ Yet, it is through the parahippocampal region (i.e. the perirhinal, parahippocampal and entorhinal cortices) that the hippocampus receives inputs and sends output to neocortical area.^111,112^ Moreover, a large body of literature has explained the parahippocampus activity as dedicated to associative memory, that is the memory that links different items together (e.g. objects, relations, places and sounds) to make it a single composite construction, namely an episodic memory.^113-116^ Thus, a parahippocampal lesion would prevent binding of the details that constitute a specific memory and thus lead to a deficit in the episodic component of ABM.

Besides the medial temporal lobe, we found, in line with other studies,^117-119^ that autobiographical memories involve the lateral temporal cortex and, particularly, the left inferior temporal gyrus (BA 20). The implication of the lateral temporal lobe would be associated with autobiographical knowledge and personal semantic memory, which feed substantially autobiographical recollection.^28^ Indeed, as proposed by Svoboda, most episodic memories contain semantic representations of what composes our environment, such as relatives, personal objects or even home. Moreover, life periods and life chapters constitute a frame for memories and provide a gateway to access more specific events.^8^ This finding in our study is particularly relevant, since the inferior temporal gyrus was correlated only in the probed recall condition, suggesting a role in the storage of information independent of retrieval abilities, as if a semanticized version of the memory was stored in these regions. The concomitant involvement of the medial temporal lobe and the lateral temporal cortex could be accounted for by the ‘transformation’ theory,^120^ which predicts that strictly episodic memories remain dependent on the medial temporal lobe and that a schematic version with fewer contextual details would develop in the neocortex as time goes by.

Among the other regions described in ABM imaging studies, we found a role for the right cerebellum, as highlighted in numerous studies that report medial and right lateralized involvement as a core neural signature of ABM, notably for its role in conscious retrieval of episodic memories by means of its connections to the dorsolateral prefrontal cortex.^101,121,122^ These results are more convincing as the cerebellum appeared to be correlated only with the free recall condition in our study, rather suggesting an involvement as an executive process. This is concordant with the existence of a strategic retrieval deficit in dementia with Lewy body patients, as attested by a higher improvement of ABM scores after probing, compared to the controls. Interestingly, we also found a correlation between the free recall condition and the putamen, which belongs to the striatum and is part of the reward circuit and has been associated with learning and episodic memory.^123,124^ Finally, our study did not reveal any correlation with the prefrontal cortex, while we might have expected it to play a role in accord with the hypothesis of an impairment in strategic retrieval. Nevertheless, we found a role for subcortical regions such as the cerebellum and the putamen, as if these regions rather than the prefrontal cortex were responsible for the executive impairments in the early stages of dementia with Lewy bodies, as is the case for attention.^125^

Overall, our study has some limitations. In particular, the sample sizes were relatively small (*n* = 20 in each group) and the results for the VBM analyses were uncorrected. Consequently, further studies involving a larger cohort of dementia with Lewy body patients will be needed to confirm our findings. Therefore, our future work aims to investigate the relationship between the subjective sense of self, the self-concept and ABM in dementia with Lewy bodies, with a special focus on the insula’s role within the different components. Moreover, we will plan to analyse the self networks with a functional neuroimaging approach.

## Conclusion

While evidence suggests that ABM impairments are a transdiagnostic feature of dementia, this study is the first to document them in dementia with Lewy bodies, from the early stage of the disease. We found that ABM impairments are temporally ungraded in dementia with Lewy body patients and are partially related to deficits in strategic retrieval processes, similar to what is described in frontotemporal lobar degeneration. The flat performance of episodic re-experiencing across life epochs, including the recent period, is also concordant with a global alteration of the self. The volumetric study suggests the involvement of posterior insular atrophy to account for the ABM deficit, as well as the involvement of the precuneus, two regions linked to elementary aspects of the self. This finding reinforces our hypothesis whereby insular damage would lead to a global collapse of the self, with consequences for autobiographical memories. Additionally, this study appears to be reliable since our results also revealed regions known to be part of the ABM network, such as the medial temporal lobe, which would bind the details of the memory, the lateral temporal lobe involved in the semantic component, the temporoparietal junction associated with the spatial context and the cerebellum, a subcortical component of strategic retrieval. Finally, the different regions identified in our study draw an ABM pattern in which the insula fits nicely, as the grounding element of the phenomenological experience.

## Supplementary Material

fcae272_Supplementary_Data

## Data Availability

The data that support the findings of this study are available from the corresponding author, upon reasonable request.

## References

[1] Filoteo JV , Salmon DP, Schiehser DM, et al Verbal learning and memory in patients with dementia with Lewy bodies or Parkinson’s disease with dementia. J Clin Exp Neuropsychol. 2009;31(7):823–834. 19221922 10.1080/13803390802572401 PMC2935683

[2] Kemp J , Philippi N, Phillipps C, et al Cognitive profile in prodromal dementia with Lewy bodies. Alz Res Therapy. 2017;9(1):19. 10.1186/s13195-017-0242-1 PMC5356316 28302161

[3] Calil V , Silveira de Souza A, Sudo FK, et al Anosognosia for memory in dementia with Lewy bodies compared with Alzheimer’s disease. Int J Geriatr Psychiatry. 2021;36(7):1059–1064. 33594752 10.1002/gps.5521

[4] Pennington C , Duncan G, Ritchie C. Altered awareness of cognitive and neuropsychiatric symptoms in Parkinson’s disease and dementia with Lewy bodies: A systematic review. Int J Geriatr Psychiatry. 2021;36(1):15–30. 32869379 10.1002/gps.5415

[5] Philippi N , Noblet V, Hamdaoui M, et al The insula, a grey matter of tastes: A volumetric MRI study in dementia with Lewy bodies. Alzheimers Res Ther. 2020;12(1):79. 32631425 10.1186/s13195-020-00645-y PMC7336457

[6] Tisserand A , Blanc F, Muller C, et al Who am I with my Lewy bodies? The insula as a core region of the self-concept networks. Alzheimers Res Ther. 2024;16(1):85. 38641653 10.1186/s13195-024-01447-2 PMC11027417

[7] Tisserand A , Noblet V, Botzung A, Blanc F, Philippi N. Who am I with my Lewy bodies? A self-concept study: Neuropsychiatry and behavioral neurology/dementia. Alzheimers Dement. 2020;16(S6).

[8] Conway MA , Pleydell-Pearce CW. The construction of autobiographical memories in the self-memory system. Psychol Rev. 2000;107(2):261–288. 10789197 10.1037/0033-295x.107.2.261

[9] Prebble SC , Addis DR, Tippett LJ. Autobiographical memory and sense of self. Psychol Bull. 2013;139(4):815–840. 23025923 10.1037/a0030146

[10] Tulving E . Memory and consciousness. Can Psychol. 1985;26:1–12.

[11] Lewis M . Ways of knowing: Objective self-awareness or consciousness. Dev Rev. 1991;11:231–243.

[12] Morin A , Everett J. Inner speech as a mediator of self-awareness, self-consciousness, and self-knowledge: An hypothesis. New Ideas Psychol. 1990;8:337–356.

[13] Singer JA . Seeing one’s self: Locating narrative memory in a framework of personality. J Pers. 1995;63(3):429–457. 7562361 10.1111/j.1467-6494.1995.tb00502.x

[14] Carver CS. Self-awareness. In: Leary MR, Tangney JP, eds. Handbook of self and identity. Guilford Press; 2012.

[15] Tisserand A , Philippi N, Botzung A, Blanc F. Me, myself and my insula: An oasis in the forefront of self-consciousness. Biology (Basel). 2023;12(4):599. 37106799 10.3390/biology12040599 PMC10135849

[16] Blanc F , Noblet V, Philippi N, et al Right anterior insula: Core region of hallucinations in cognitive neurodegenerative diseases. PLoS One. 2014;9(12):e114774. 25479196 10.1371/journal.pone.0114774 PMC4257732

[17] Blanc F , Colloby SJ, Philippi N, et al Cortical thickness in dementia with Lewy bodies and Alzheimer’s disease: A comparison of prodromal and dementia stages. PLoS One. 2015;10(6):e0127396. 26061655 10.1371/journal.pone.0127396 PMC4489516

[18] Blanc F , Colloby SJ, Cretin B, et al Grey matter atrophy in prodromal stage of dementia with Lewy bodies and Alzheimer’s disease. Alz Res Therapy. 2016;8(1):31. 10.1186/s13195-016-0198-6 PMC4970221 27484179

[19] Roquet D , Noblet V, Anthony P, et al Insular atrophy at the prodromal stage of dementia with Lewy bodies: A VBM DARTEL study. Sci Rep. 2017;7(1):9437. 28842567 10.1038/s41598-017-08667-7 PMC5573371

[20] Craig AD . How do you feel–now? The anterior insula and human awareness. Nat Rev Neurosci. 2009;10(1):59–70. 19096369 10.1038/nrn2555

[21] Damasio A . Feelings of emotion and the self. Ann N Y Acad Sci. 2003;1001:253–261. 14625365 10.1196/annals.1279.014

[22] Modinos G , Ormel J, Aleman A. Activation of anterior insula during self-reflection. PLoS One. 2009;4(2):4618. 10.1371/journal.pone.0004618 PMC2643476 19242539

[23] Conway MA . Sensory–perceptual episodic memory and its context: Autobiographical memory. Phil Trans R Soc Lond B. 2001;356(1413):1375–1384. 11571029 10.1098/rstb.2001.0940 PMC1088521

[24] Conway MA . Memory and the self. J Mem Lang. 2005;53(4):594–628.

[25] Levine B , Svoboda E, Hay JF, Winocur G, Moscovitch M. Aging and autobiographical memory: Dissociating episodic from semantic retrieval. Psychol Aging. 2002;17(4):677–689. 12507363

[26] Gallagher II . Philosophical conceptions of the self: Implications for cognitive science. Trends Cogn Sci. 2000;4(1):14–21. 10637618 10.1016/s1364-6613(99)01417-5

[27] Neisser U . Five kinds of self-knowledge. Philos Psychol. 1988;1(1):35–59.

[28] Svoboda E , McKinnon MC, Levine B. The functional neuroanatomy of autobiographical memory: A meta-analysis. Neuropsychologia. 2006;44(12):2189–2208. 16806314 10.1016/j.neuropsychologia.2006.05.023 PMC1995661

[29] Fink GR , Markowitsch HJ, Reinkemeier M, Bruckbauer T, Kessler J, Heiss WD. Cerebral representation of one’s own past: Neural networks involved in autobiographical memory. J Neurosci. 1996;16(13):4275–4282. 8753888 10.1523/JNEUROSCI.16-13-04275.1996 PMC6579004

[30] Descamps M , Boucher O, Nguyen DK, Rouleau I. Emotional autobiographical memory associated with insular resection in epileptic patients: A comparison with temporal lobe resection. Brain Sci. 2021;11(10):1316. 34679381 10.3390/brainsci11101316 PMC8533905

[31] Berna F , Potheegadoo J, Aouadi I, et al A meta-analysis of autobiographical memory studies in schizophrenia spectrum disorder. Schizophr Bull. 2016;42(1):56–66. 26209548 10.1093/schbul/sbv099 PMC4681554

[32] Wantzen P , Boursette A, Zante E, et al Autobiographical memory and social identity in autism: Preliminary results of social positioning and cognitive intervention. Front Psychol. 2021;12:641765. 33815227 10.3389/fpsyg.2021.641765 PMC8009988

[33] Irish M . Autobiographical memory in dementia syndromes—An integrative review. Wiley Interdiscip Rev Cogn Sci. 2023;14(3):e1630. 36239297 10.1002/wcs.1630

[34] Addis DR , Sacchetti DC, Ally BA, Budson AE, Schacter DL. Episodic simulation of future events is impaired in mild Alzheimer’s disease. Neuropsychologia. 2009;47(12):2660–2671. 19497331 10.1016/j.neuropsychologia.2009.05.018 PMC2734895

[35] Piolino P . Autobiographical memory and autonoetic consciousness: Triple dissociation in neurodegenerative diseases. Brain. 2003;126(10):2203–2219. 12821510 10.1093/brain/awg222

[36] Irish M , Lawlor BA, O'Mara SM, Coen RF. Impaired capacity for autonoetic reliving during autobiographical event recall in mild Alzheimer’s disease. Cortex. 2011;47(2):236–249. 20153463 10.1016/j.cortex.2010.01.002

[37] Philippi N , Noblet V, Botzung A, et al MRI-based volumetry correlates of autobiographical memory in Alzheimer’s disease. PLoS One. 2012;7(10):46200. 10.1371/journal.pone.0046200 PMC3468599 23071546

[38] Philippi N , Rousseau F, Noblet V, et al Different temporal patterns of specific and general autobiographical memories across the lifespan in Alzheimer’s disease. Behav Neurol. 2015;2015:963460. 26175549 10.1155/2015/963460 PMC4484842

[39] Mion M , Patterson K, Acosta-Cabronero J, et al What the left and right anterior fusiform gyri tell us about semantic memory. Brain. 2010;133(11):3256–3268. 20952377 10.1093/brain/awq272

[40] Irish M , Hornberger M, Lah S, et al Profiles of recent autobiographical memory retrieval in semantic dementia, behavioural-variant frontotemporal dementia, and Alzheimer’s disease. Neuropsychologia. 2011;49(9):2694–2702. 21658396 10.1016/j.neuropsychologia.2011.05.017

[41] Graham KS , Hodges JR. Differentiating the roles of the hippocampus complex and the neocortex in long-term memory storage: Evidence from the study of semantic dementia and Alzheimer’s disease. Neuropsychology. 1997;11(1):77–89. 9055272 10.1037//0894-4105.11.1.77

[42] Rabinovici GD , Seeley WW, Kim EJ, et al Distinct MRI atrophy patterns in autopsy-proven Alzheimer’s disease and frontotemporal lobar degeneration. Am J Alzheimers Dis Other Demen. 2008;22(6):474–488. 10.1177/1533317507308779 PMC2443731 18166607

[43] Seeley WW . Selective functional, regional, and neuronal vulnerability in frontotemporal dementia. Curr Opin Neurol. 2008;21(6):701–707. 18989116 10.1097/WCO.0b013e3283168e2d PMC2909835

[44] Irish M , Landin-Romero R, Mothakunnel A, et al Evolution of autobiographical memory impairments in Alzheimer’s disease and frontotemporal dementia—A longitudinal neuroimaging study. Neuropsychologia. 2018;110:14–25. 28288787 10.1016/j.neuropsychologia.2017.03.014

[45] Ahmed S , Irish M, Loane C, et al Association between precuneus volume and autobiographical memory impairment in posterior cortical atrophy: Beyond the visual syndrome. NeuroImage Clin. 2018;18:822–834. 29876268 10.1016/j.nicl.2018.03.008 PMC5988022

[46] McKeith I , Dickson DW, Lowe J, et al Diagnosis and management of dementia with Lewy bodies: Third report of the DLB consortium. Neurology. 2005;65(12):1863–1872. 16237129 10.1212/01.wnl.0000187889.17253.b1

[47] Zaccai J , McCracken C, Brayne C. A systematic review of prevalence and incidence studies of dementia with Lewy bodies. Age Ageing. 2005;34(6):561–566. 16267179 10.1093/ageing/afi190

[48] McKeith IG , Ferman TJ, Thomas AJ, et al Research criteria for the diagnosis of prodromal dementia with Lewy bodies. Neurology. 2020;94(17):743–755. 32241955 10.1212/WNL.0000000000009323 PMC7274845

[49] McKeith IG , Boeve BF, Dickson DW, et al Diagnosis and management of dementia with Lewy bodies: Fourth consensus report of the DLB Consortium. Neurology. 2017;89(1):88–100. 28592453 10.1212/WNL.0000000000004058 PMC5496518

[50] Lawton MP , Brody EM. Assessment of older people: Self-maintaining and instrumental activities of daily living. Gerontologist. 1969;9(3):179–186. 5349366

[51] American Psychiatric Association, American Psychiatric Association . Diagnostic and statistical manual of mental disorders: DSM-5. 5th edn. American Psychiatric Association; 2013.

[52] Ferman TJ , Smith GE, Boeve BF, et al DLB fluctuations: Specific features that reliably differentiate DLB from AD and normal aging. Neurology. 2004;62(2):181–187. 14745051 10.1212/wnl.62.2.181

[53] Walker MP , Ayre GA, Cummings JL, et al The clinician assessment of fluctuation and the one day fluctuation assessment scale: Two methods to assess fluctuating confusion in dementia. Br J Psychiatry. 2000;177(3):252–256. 11040887 10.1192/bjp.177.3.252

[54] Fénelon G , Soulas T, Zenasni F, de Langavant LC. The changing face of Parkinson’s disease-associated psychosis: A cross-sectional study based on the new NINDS-NIMH criteria. Mov Disord. 2010;25(6):763–766. 20437542 10.1002/mds.22839 PMC2891710

[55] Gjerstad MD , Boeve B, Wentzel-Larsen T, Aarsland D, Larsen JP. Occurrence and clinical correlates of REM sleep behaviour disorder in patients with Parkinson’s disease over time. J Neurol Neurosurg Psychiatry. 2008;79(4):387–391. 17557796 10.1136/jnnp.2007.116830

[56] Clément JP , Nassif RF, Léger JM, Marchan F. Development and contribution to the validation of a brief French version of the Yesavage Geriatric Depression Scale. Encephale. 1997;23(2):91–99. 9264935

[57] Hachinski VC , Iliff LD, Zilhka E, et al Cerebral blood flow in dementia. Arch Neurol. 1975;32(9):632–637. 1164215 10.1001/archneur.1975.00490510088009

[58] Van der Linden M , Coyette F, Poitrenaud J, et al L’épreuve de rappel libre/rappel indicé à 16 items (RL/RI-16). In: L’évaluation Des Troubles de La Mémoire: Présentation de Quatre Tests de Mémoire Épisodique (Avec Leur Étalonnage). Solal; 2004.

[59] Grober E , Buschke H, Crystal H, Bang S, Dresner R. Screening for dementia by memory testing. Neurology. 1988;38(6):900–900. 3368071 10.1212/wnl.38.6.900

[60] Thurstone L , Thurstone T. Manuel d’application de La Batterie Factorielle P.M.A (primary mental activities). 2nd edn. Adaptation Française du Centre de Psychologie Appliquée; 1964.

[61] Dubois B , Slachevsky A, Litvan I, Pillon B. The FAB: A frontal assessment battery at bedside. Neurology. 2000;55(11):1621–1626. 11113214 10.1212/wnl.55.11.1621

[62] Goul WR , Brown M. Effects of age and intelligence on trail making test performance and validity. Percept Mot Skills. 1970;30(1):319–326. 5476120 10.2466/pms.1970.30.1.319

[63] Wechsler D . Wechsler adult intelligence scale. 3rd edn. The Psychological Corporation; 1997.

[64] Osterrieth PA. Le test de copie d’une figure complexe; contribution à l’étude de la perception et de la mémoire [Test of copying a complex figure; contribution to the study of perception and memory]. Arch Psychol. 1944; 30:206–356.

[65] Rosenbaum RS , Moscovitch M, Foster JK, et al Patterns of autobiographical memory loss in medial-temporal lobe amnesic patients. J Cogn Neurosci. 2008;20(8):1490–1506. 18303977 10.1162/jocn.2008.20105

[66] Verfaellie M , Bousquet K, Keane MM. Medial temporal and neocortical contributions to remote memory for semantic narratives: Evidence from amnesia. Neuropsychologia. 2014;61:105–112. 24953960 10.1016/j.neuropsychologia.2014.06.018 PMC4122606

[67] Good CD , Johnsrude IS, Ashburner J, Henson RNA, Friston KJ, Frackowiak RSJ. A voxel-based morphometric study of ageing in 465 normal adult human brains. NeuroImage. 2001;14(1):21–36. 11525331 10.1006/nimg.2001.0786

[68] Ashburner J , Friston KJ. Unified segmentation. NeuroImage. 2005;26(3):839–851. 15955494 10.1016/j.neuroimage.2005.02.018

[69] Kantarci K , Nedelska Z, Chen Q, et al Longitudinal atrophy in prodromal dementia with Lewy bodies points to cholinergic degeneration. Brain Commun. 2022;4(2):fcac013. 35415608 10.1093/braincomms/fcac013 PMC8994111

[70] Wheeler MA , Stuss DT, Tulving E. Toward a theory of episodic memory: The frontal lobes and autonoetic consciousness. Psychol Bull. 1997;121(3):331–354. 9136640 10.1037/0033-2909.121.3.331

[71] Craig AD . Interoception: The sense of the physiological condition of the body. Curr Opin Neurobiol. 2003;13(4):500–505. 12965300 10.1016/s0959-4388(03)00090-4

[72] Cavalcante LES , Zinn CG, Schmidt SD, et al Modulation of the storage of social recognition memory by neurotransmitter systems in the insular cortex. Behav Brain Res. 2017;334:129–134. 28760699 10.1016/j.bbr.2017.07.044

[73] Guzmán-Ramos K , Bermúdez-Rattoni F. Interplay of amygdala and insular cortex during and after associative taste aversion memory formation. Rev Neurosci. 2012;23(5-6):5–6. 10.1515/revneuro-2012-0056 23001315

[74] Bermudez-Rattoni F . The forgotten insular cortex: Its role on recognition memory formation. Neurobiol Learn Mem. 2014;109:207–216. 24406466 10.1016/j.nlm.2014.01.001

[75] Bermudez-Rattoni F , Okuda S, Roozendaal B, McGaugh JL. Insular cortex is involved in consolidation of object recognition memory. Learn Mem. 2005;12(5):447–449. 16166398 10.1101/lm.97605

[76] Paller KA , Ranganath C, Gonsalves B, et al Neural correlates of person recognition. Learn Mem. 2003;10(4):253–260. 12888543 10.1101/lm.57403 PMC202315

[77] Karnath HO , Baier B, Nagele T. Awareness of the functioning of one’s own limbs mediated by the insular cortex? J Neurosci. 2005;25(31):7134–7138. 16079395 10.1523/JNEUROSCI.1590-05.2005 PMC6725240

[78] Kletenik I , Gaudet K, Prasad S, Cohen AL, Fox MD. Network localization of awareness in visual and motor anosognosia. Ann Neurol. 2023;94(3):434–441. 37289520 10.1002/ana.26709 PMC10524951

[79] Philippi N , Roquet D, Ben Malek H, et al Henry, where have you lost your self? Cortex. 2017;95:37–50. 28843132 10.1016/j.cortex.2017.06.019

[80] Critchley HD , Wiens S, Rotshtein P, Ohman A, Dolan RJ. Neural systems supporting interoceptive awareness. Nat Neurosci. 2004;7(2):189–195. 14730305 10.1038/nn1176

[81] Devue C , Collette F, Balteau E, et al Here I am: The cortical correlates of visual self-recognition. Brain Res. 2007;1143:169–182. 17306235 10.1016/j.brainres.2007.01.055

[82] Gusnard DA , Akbudak E, Shulman GL, Raichle ME. Medial prefrontal cortex and self-referential mental activity: Relation to a default mode of brain function. Proc Natl Acad Sci U S A. 2001;98(7):4259–4264. 11259662 10.1073/pnas.071043098 PMC31213

[83] Kircher TT , Senior C, Phillips ML, et al Towards a functional neuroanatomy of self processing: Effects of faces and words. Brain Res Cogn Brain Res. 2000;10(1–2):133–144. 10978701 10.1016/s0926-6410(00)00036-7

[84] Baier B , Karnath HO. Tight link between our sense of limb ownership and self-awareness of actions. Stroke. 2008;39(2):486–488. 18162622 10.1161/STROKEAHA.107.495606

[85] Farrer C , Franck N, Georgieff N, Frith CD, Decety J, Jeannerod M. Modulating the experience of agency: A positron emission tomography study. NeuroImage. 2003;18(2):324–333. 12595186 10.1016/s1053-8119(02)00041-1

[86] Schulz SM . Neural correlates of heart-focused interoception: a functional magnetic resonance imaging meta-analysis. Philos Trans R Soc Lond B Biol Sci. 2016;371(1708):20160018. 28080975 10.1098/rstb.2016.0018 PMC5062106

[87] Stephan E , Pardo JV, Faris PL, et al Functional neuroimaging of gastric distention. J Gastrointest Surg. 2003;7(6):740–749. 13129550 10.1016/s1091-255x(03)00071-4

[88] Tsakiris M , Hesse MD, Boy C, Haggard P, Fink GR. Neural signatures of body ownership: A sensory network for bodily self-consciousness. Cereb Cortex. 2007;17(10):2235–2244. 17138596 10.1093/cercor/bhl131

[89] Northoff G , Heinzel A, Greck M, Bermpohl F, Dobrowolny H, Panksepp J. Self-referential processing in our brain—A meta-analysis of imaging studies on the self. Neuroimage. 2006;31(1):440–457. 16466680 10.1016/j.neuroimage.2005.12.002

[90] Damasio A , Meyer K. Consciousness: An overview of the phenomenon and of its possible neural basis. In: The neurology of consciousness. Elsevier; 2009:3–14. doi:10.1016/B978-0-12-374168-4.00001-0

[91] Howe ML , Courage ML. The emergence and early development of autobiographical memory. Psychol Rev. 1997;104(3):499–523. 9243962 10.1037/0033-295x.104.3.499

[92] Northoff G , Bermpohl F. Cortical midline structures and the self. Trends Cogn Sci. 2004;8(3):102–107. 15301749 10.1016/j.tics.2004.01.004

[93] Farrer C , Frith CD. Experiencing oneself vs another person as being the cause of an action: The neural correlates of the experience of agency. Neuroimage. 2002;15(3):596–603. 11848702 10.1006/nimg.2001.1009

[94] Lyu D , Stieger JR, Xin C, et al Causal evidence for the processing of bodily self in the anterior precuneus. Neuron. 2023;111(16):2502–2512.e4. 37295420 10.1016/j.neuron.2023.05.013

[95] Ruby P , Decety J. Effect of subjective perspective taking during simulation of action: A PET investigation of agency. Nat Neurosci. 2001;4(5):546–550. 11319565 10.1038/87510

[96] Farrow TF , Zheng Y, Wilkinson ID, et al Investigating the functional anatomy of empathy and forgiveness. Neuroreport. 2001;12(11):2433–2438. 11496124 10.1097/00001756-200108080-00029

[97] Kircher TTJ , Brammer M, Bullmore E, Simmons A, Bartels M, David AS. The neural correlates of intentional and incidental self processing. Neuropsychologia. 2002;40(6):683–692. 11792407 10.1016/s0028-3932(01)00138-5

[98] Kjaer TW , Nowak M, Lou HC. Reflective self-awareness and conscious states: PET evidence for a common midline parietofrontal core. NeuroImage. 2002;17(2):1080–1086. 12377180

[99] Lou HC , Luber B, Crupain M, et al Parietal cortex and representation of the mental self. Proc Natl Acad Sci U S A. 2004;101(17):6827–6832. 15096584 10.1073/pnas.0400049101 PMC404216

[100] Addis DR , McIntosh AR, Moscovitch M, Crawley AP, McAndrews MP. Characterizing spatial and temporal features of autobiographical memory retrieval networks: A partial least squares approach. Neuroimage. 2004;23(4):1460–1471. 15589110 10.1016/j.neuroimage.2004.08.007

[101] Gilboa A . Autobiographical and episodic memory—One and the same? Evidence from prefrontal activation in neuroimaging studies. Neuropsychologia. 2004;42(10):1336–1349. 15193941 10.1016/j.neuropsychologia.2004.02.014

[102] Fossati P . Imaging autobiographical memory. Dialogues Clin Neurosci. 2013;15(4):487–490. 24459415 10.31887/DCNS.2013.15.4/pfossati PMC3898686

[103] Fletcher PC , Frith CD, Baker SC, Shallice T, Frackowiak RS, Dolan RJ. The mind’s eye—Precuneus activation in memory-related imagery. Neuroimage. 1995;2(3):195–200. 9343602 10.1006/nimg.1995.1025

[104] Freton M , Lemogne C, Bergouignan L, Delaveau P, Lehéricy S, Fossati P. The eye of the self: Precuneus volume and visual perspective during autobiographical memory retrieval. Brain Struct Funct. 2014;219(3):959–968. 23553546 10.1007/s00429-013-0546-2

[105] Burgess N , Maguire EA, O’Keefe J. The human hippocampus and spatial and episodic memory. Neuron. 2002; 35(4):625–641. 12194864 10.1016/s0896-6273(02)00830-9

[106] Nadel L , Campbell J, Ryan L. Autobiographical memory retrieval and hippocampal activation as a function of repetition and the passage of time. Neural Plast. 2007;2007:1–14. 10.1155/2007/90472 PMC2233815 18274617

[107] Gilboa A , Ramirez J, Kohler S, Westmacott R, Black SE, Moscovitch M. Retrieval of autobiographical memory in Alzheimer’s disease: Relation to volumes of medial temporal lobe and other structures. Hippocampus. 2005;15(4):535–550. 15884035 10.1002/hipo.20090

[108] Scoville WB , Milner B. Loss of recent memory after bilateral hippocampal lesions. J Neurol Neurosurg Psychiatry. 1957;20(1):11–21. 13406589 10.1136/jnnp.20.1.11 PMC497229

[109] Squire LR . Memory and the hippocampus: A synthesis from findings with rats, monkeys, and humans. Psychol Rev. 1992;99(2):195–231. 1594723 10.1037/0033-295x.99.2.195

[110] Adamowicz DH , Roy S, Salmon DP, et al Hippocampal α-synuclein in dementia with Lewy bodies contributes to memory impairment and is consistent with spread of pathology. J Neurosci. 2017;37(7):1675–1684. 28039370 10.1523/JNEUROSCI.3047-16.2016 PMC5320602

[111] Eichenbaum H , Lipton PA. Towards a functional organization of the medial temporal lobe memory system: Role of the parahippocampal and medial entorhinal cortical areas. Hippocampus. 2008;18(12):1314–1324. 19021265 10.1002/hipo.20500 PMC2592493

[112] Squire LR , Stark CEL, Clark RE. The medial temporal lobe. Annu Rev Neurosci. 2004;27(1):279–306. 15217334 10.1146/annurev.neuro.27.070203.144130

[113] Davachi L , Mitchell JP, Wagner AD. Multiple routes to memory: Distinct medial temporal lobe processes build item and source memories. Proc Natl Acad Sci USA. 2003;100(4):2157–2162. 12578977 10.1073/pnas.0337195100 PMC149975

[114] Eacott MJ , Gaffan EA. The roles of perirhinal cortex, postrhinal cortex, and the fornix in memory for objects, contexts, and events in the rat. Q J Exp Psychol B. 2005;58(3–4):202–217. 16194965 10.1080/02724990444000203

[115] Kirwan CB , Stark CEL. Medial temporal lobe activation during encoding and retrieval of novel face-name pairs. Hippocampus. 2004;14(7):919–930. 15382260 10.1002/hipo.20014 PMC2704554

[116] Yang J , Meckingler A, Xu M, Zhao Y, Weng X. Decreased parahippocampal activity in associative priming: Evidence from an event-related fMRI study. Learn Mem. 2008;15(9):703–710. 18772259 10.1101/lm.900108 PMC2722909

[117] Gilboa A . Remembering our past: Functional neuroanatomy of recollection of recent and very remote personal events. Cerebral Cortex. 2004;14(11):1214–1225. 15166099 10.1093/cercor/bhh082

[118] Levine B , Turner GR, Tisserand D, Hevenor SJ, Graham SJ, McIntosh AR. The functional neuroanatomy of episodic and semantic autobiographical remembering: A prospective functional MRI study. J Cogn Neurosci. 2004;16(9):1633–1646. 15601525 10.1162/0898929042568587

[119] Maguire EA . Aging affects the engagement of the hippocampus during autobiographical memory retrieval. Brain. 2003;126(7):1511–1523. 12805116 10.1093/brain/awg157

[120] Winocur G , Moscovitch M, Bontempi B. Memory formation and long-term retention in humans and animals: Convergence towards a transformation account of hippocampal–neocortical interactions. Neuropsychologia. 2010;48(8):2339–2356. 20430044 10.1016/j.neuropsychologia.2010.04.016

[121] Andreasen NC , O’Leary DS, Paradiso S, et al The cerebellum plays a role in conscious episodic memory retrieval. Hum Brain Mapp. 1999;8(4):226–234. 10619416 10.1002/(SICI)1097-0193(1999)8:4<226::AID-HBM6>3.0.CO;2-4 PMC6873320

[122] Middleton FA , Strick PL. Anatomical evidence for cerebellar and basal ganglia involvement in higher cognitive function. Science. 1994;266(5184):458–461. 7939688 10.1126/science.7939688

[123] Brickman AM , Buchsbaum MS, Shihabuddin L, Hazlett EA, Borod JC, Mohs RC. Striatal size, glucose metabolic rate, and verbal learning in normal aging. Brain Res Cogn Brain Res. 2003;17(1):106–116. 12763197 10.1016/s0926-6410(03)00085-5

[124] Cervenka S , Bäckman L, Cselényi Z, Halldin C, Farde L. Associations between dopamine D2-receptor binding and cognitive performance indicate functional compartmentalization of the human striatum. NeuroImage. 2008;40(3):1287–1295. 18296072 10.1016/j.neuroimage.2007.12.063

[125] Botzung A , Philippi N, Noblet V, Loureiro De Sousa P, Blanc F. Pay attention to the basal ganglia: A volumetric study in early dementia with Lewy bodies. Alz Res Therapy. 2019;11(1):108. 10.1186/s13195-019-0568-y PMC6925479 31864422

