# Supplementary material for: Neuroimaging of autobiographical memory in dementia with Lewy bodies: a story of insula
Source: Brain Commun. 2024 Aug 20;6(4):fcae272. doi: 10.1093/braincomms/fcae272 (PMC11358644; doi:10.1093/braincomms/fcae272)
Supplement: fcae272_Supplementary_Data [file fcae272_supplementary_data.pdf]

Supplementary table 1

Mean scores on neuropsychological tests in patients with dementia with Lewy bodies

Neuropsychological evaluation	RL/RI16						FAB	Semantic fluency	Phonological fluency	TMTA		TMTB		Digit Span	ROCF
	RL1	*RT1*	*RL2*	*RT2*	*RL3*	*RT3*				*time*	*errors*	*time*	*errors*		
**Patients mean score (SD)**	5.1* (2.53)	11.4* (3.82)	5.75* (3.49)	12.2* (3.9)	6.05* (3.32)	12.7* (4.33)	14.11* (2.88)	20.50 (6.44)	17.25 (7.47)	72.68 (38.15	0.26* (0.56)	140.78 (68.71)	0.78 (1.05)	10.1 (2.99)	28.15* (9.09)
**Normative data mean score (SD)**	8.4 (2.5)	14.3 (2.1)	9.2 (2.4)	14.8 (1.8)	11.2 (2.8)	15.6 (0.8)	17.3 (0.8)	26.8 (7.4)	19.7 (6.7)	60,08 (26,76)	0 (0)	118.50 (50.67)	1 (1.35)	9 (cutoff)	31.38 (4)

FAB Frontal Assessment Battery**; RL/RI16** Rappel libre/Rappel indicé à 16 items**; ROCF** Rey-Osterrieth Complex Figure; **TMT** Trail Making Test. Data were available for 20 patients for the RL/RI16, semantic and phonological fluencies and digit span, for 19 patients for the BREF, TMTA and ROCF, and for 14 patients for the TMTB.

Scores marked with a star represent impaired scores, according to normative data.

Supplementary table 2

VBM results for the free recall and probed recall conditions on the Autobiographical Interview in the dementia with Lewy bodies group

Cluster	VBM	Side	BA	*k*		*X*	*y*	*z*	T
				FRS	***PRS***				
1.	Posterior insular cortex	R	13	216/813	226/765	46.5	-27	18	3.82
	Temporoparietal junction	R	41	361/813	305/765	48	-30	21	3.74
2.	Precuneus	R	7	97/112	184/194	19.5	-72	43.5	4.48
3.	Parahippocampal gyrus	R	28/36	120/126	509/544	19.5	-3	-31.5	3.55
4.	Parahippocampal gyrus	L	35/36	393/701	423/709	-24	-21	-28.5	3.95
	Cerebellum	L	NA	109/701	104/709	-31.5	-39	-25.5	3.28
5.	Cerebellum	R	NA	93/100	-	36	-43.5	-25.5	3.43
6.	Putamen	R	NA	92/195	-	30	-7.5	-6	3.40
7.	Inferior temporal gyrus	L	20	-	77/137	-52.5	-22.5	-31.5	3.43

FRS free recall score, *PRS* probed recall score, *L* left, *R* right, *BA* Brodmann area, *k* cluster size in voxels (specific region's volume/cluster's global volume), x, y, z Talairach coordinates, T T-value

Supplementary figure 1

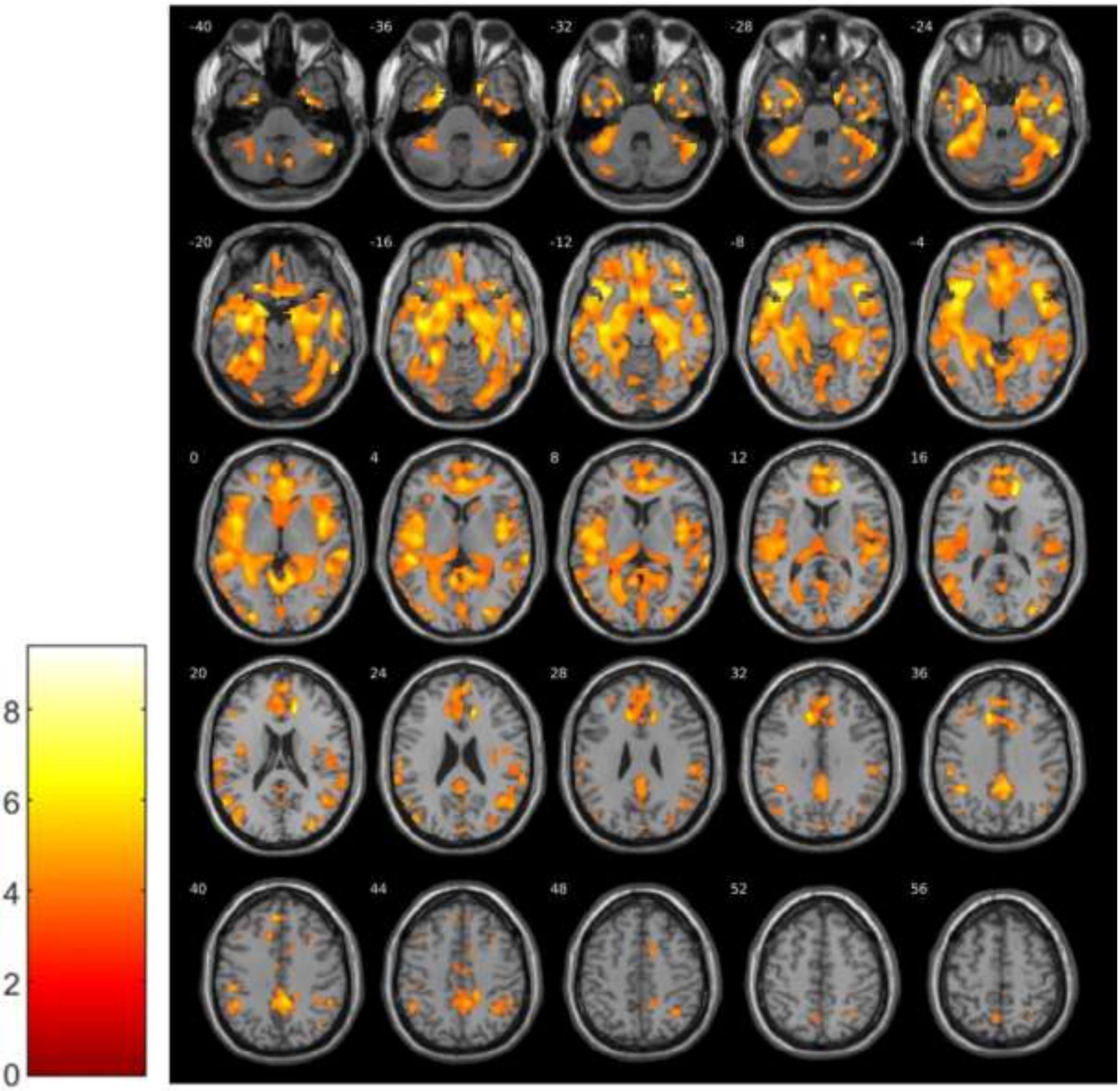

VBM analyses in LB patients compared to healthy controls. Two sample *t* test was used to compare grey matter volume in DLB patients ($n = 20$) versus control subjects ($n = 20$). Cortical thinning involving insular, temporal, occipital, frontal, cingular cortices and to a lesser extent parietal cortex in LB patients compared to healthy controls, including TIV and age as nuisance covariates ($p < .05$, FDR corrected)

Supplementary figure 2

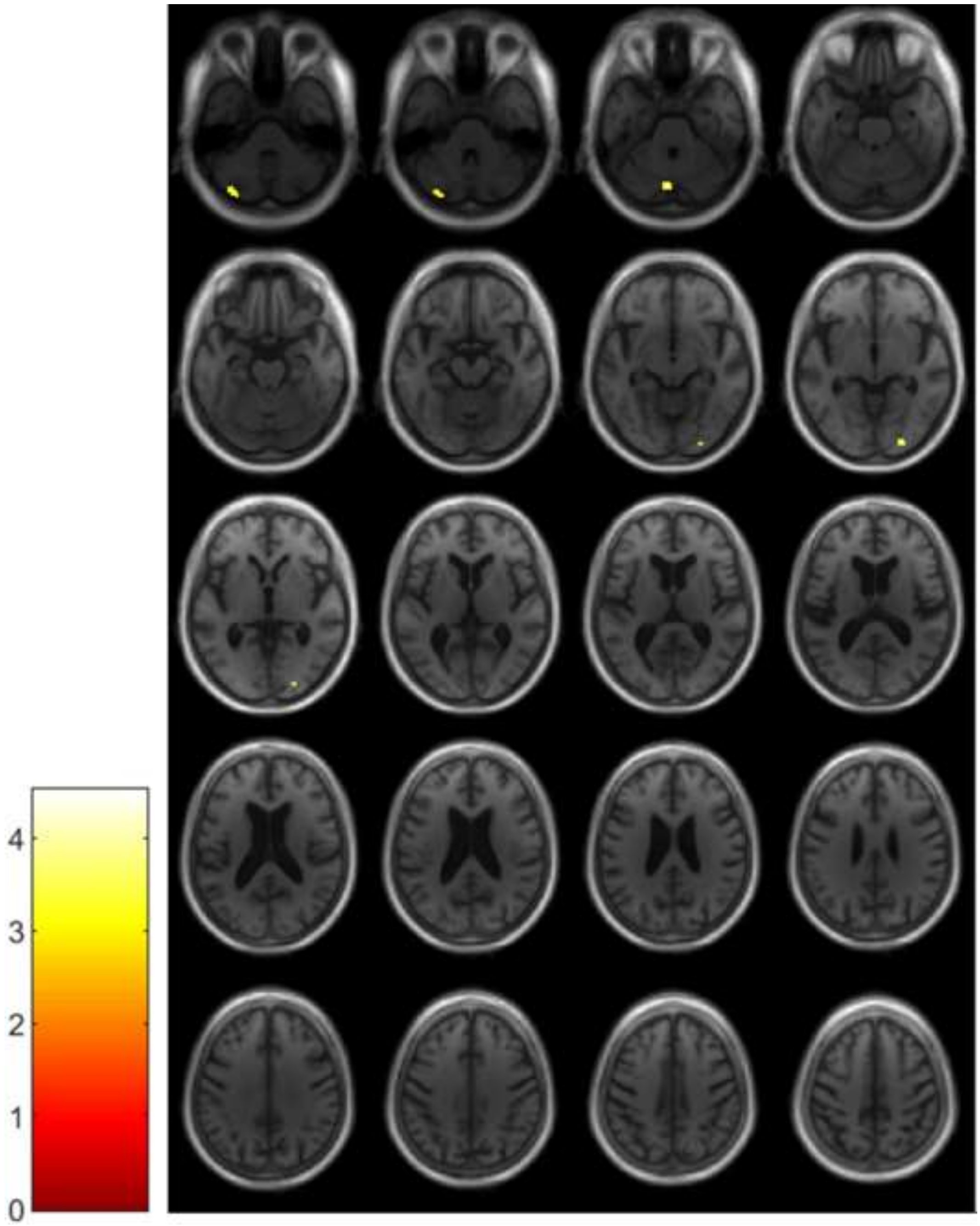

VBM analyses for RL/RI16 1-3 free recall condition in the dementia with Lewy bodies group. Multiple linear regression was used to obtain correlation for RL/RI-16 1-3 free recall score and grey matter volume in DLB patients (*n* = 20). Grey matter volume within cerebellum is positively correlated with the 1-3 free recall score on the RL/RI16, using a threshold of *P* = .005 uncorrected, including age, gender, TIV and MMSE score as nuisance covariates, *k* = 50.

Supplementary figure 3

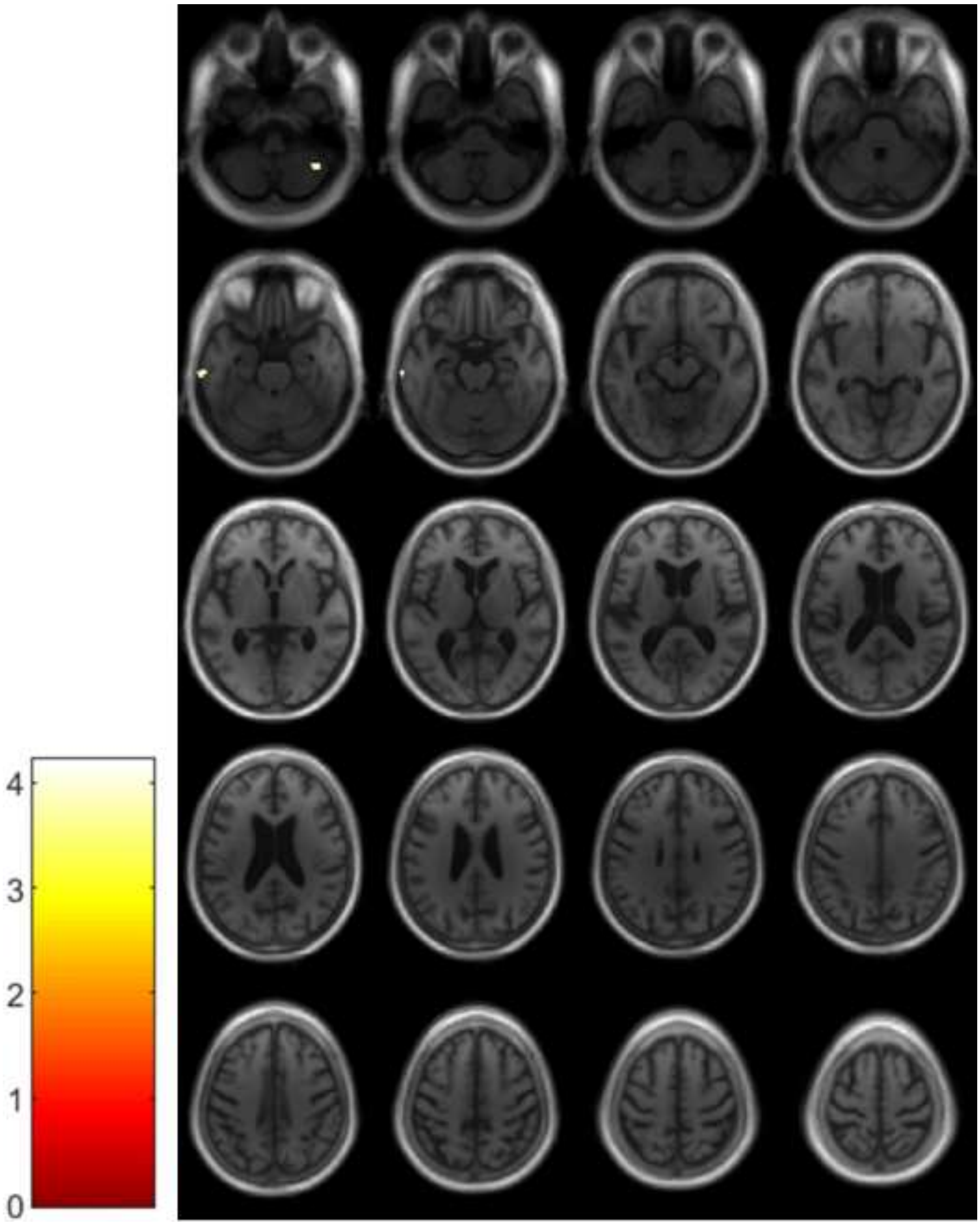

VBM analyses for RL/RI16 1-3 total recall condition in the dementia with Lewy bodies group. Multiple linear regression was used to obtain correlation for RL/RI-16 1-3 total recall score and grey matter volume in DLB patients (*n* = 20). Grey matter volumes within cerebellum and left lateral temporal lobe are positively correlated with the 1-3 total recall score on the RL/RI16, using a threshold of *P* = .005 uncorrected, including age, gender, TIV and MMSE score as nuisance covariates, *k* = 50.
